# Glycemic variability in patients with Wolfram syndrome is lower than in type 1 diabetes

**DOI:** 10.1007/s00592-015-0757-5

**Published:** 2015-04-29

**Authors:** A. Zmyslowska, W. Fendler, A. Szadkowska, M. Borowiec, M. Mysliwiec, A. Baranowska-Jazwiecka, M. Buraczewska, M. Fulmanska-Anders, B. Mianowska, I. Pietrzak, D. Rzeznik, W. Mlynarski

**Affiliations:** Department of Pediatrics, Oncology, Hematology and Diabetology, Medical University of Lodz, Sporna Str. 36/50, 91-738 Lodz, Poland; Department of Clinical Genetics, Medical University of Lodz, Lodz, Poland; Department of Pediatrics, Diabetology and Endocrinology, Medical University of Gdansk, Gdańsk, Poland

**Keywords:** Continuous glucose monitoring system, Wolfram syndrome, GlyCulator, Type 1 diabetes

## Abstract

**Aims:**

Wolfram syndrome (WFS) is diagnosed as coexistence of diabetes mellitus and optic atrophy, where pancreatic beta cell destruction is associated with neurodegeneration. Typically, WFS necessitates insulin treatment similar to type 1 diabetes (T1D), but the mechanism of beta cell mass reduction leading to hyperglycemia is different.

**Methods:**

The aim of the study was to assess glycemic variability using the continuous glucose monitoring (CGM) system in seven pediatric patients with genetically confirmed WFS and compare the results with data obtained from 21 propensity score-matched patients with T1D. The “GlyCulator” application was used for the calculation of glycemic variability indices.

**Results:**

CGM recordings showed similarities in glycemic variability among WFS patients, but differing from those of the T1D group. Coefficient of variation (%CV), CONGA4h, and GONGA6h were significantly (*p* < 0.05) lower in WFS patients (28.08 ± 7.37, 54.96 ± 11.92, and 55.99 ± 10.58) than in T1D patients (37.87 ± 14.24, 74.12 ± 28.74, *p* = 0.02, and 80.26 ± 35.05, respectively). In WFS patients, the percentage of values above 126 mg/dL was 69.79 (52.08–77.43), whereas in patients with T1D, the percentage was significantly lower—47.22 (35.07–62.85, *p* = 0.018). Curiously, a tendency toward a lower percentage of measurements below 70 mg/dL was noted in the WFS group [0 (0–7.29)] in comparison with the T1D group [6.25 (0–18.06), *p* = 0.122]. WFS patients had a significantly higher C-peptide level (0.31 ± 0.2 ng/mL) than T1D patients (0.04 ± 0.04 ng/mL; *p* = 0.006).

**Conclusions:**

Patients with WFS show smaller glycemic variability than individuals with T1D, and this may be associated with persistent residual insulin secretion.

## Introduction

The continuous glucose monitoring (CGM) system is a very useful tool in the management of patients with insulin-treated diabetes, particularly type 1 diabetes (T1D) [1]. The system was developed to assess glycemic variability with the aim of improving the degree of metabolic control of diabetes to reduce the risk of both chronic complications and episodes of hypoglycemia [2, 3]. Recent studies have confirmed the beneficial effects of CGM not only on metabolic control, but also on overall treatment satisfaction and quality of life of children with T1D [4, 5]. Many attempts have also been made to use CGM to predict diabetes mellitus in children with hyperglycemia [6, 7]. Interestingly, CGM can also be helpful in differentiating between types of diabetes, such as in patients with monogenic diabetes [8].

One of the syndromic forms of monogenic diabetes in children and adolescents is the autosomal recessively inherited Wolfram syndrome (WFS), for which the clinical criteria have been clearly defined as coexistence of diabetes mellitus and optic atrophy. Because WFS is a neurodegenerative disorder, most WFS patients suffer also from diabetes insipidus, deafness, and many other multi-organ abnormalities [9, 10]. Diabetes in WFS patients is non-autoimmune type of diabetes [9]. However, it is associated with insulin deficiency due to selective loss of pancreatic β-cells and impaired insulin secretion [10, 11]. Studies performed in animal models confirmed that wolframin—the product of the *WFS*-*1* gene—is selectively produced by the β-cells of the pancreas, and islets deprived of its expression are more prone to apoptosis [12, 13]. To date, the studies that have characterized the course of diabetes in WFS patients have shown inconsistent results concerning the degree of metabolic control (based on HbA1c level) and insulin requirement in comparison with T1D patients [14, 15].

The aim of the present study was to evaluate glycemic variability using the CGM system in pediatric patients with insulin-treated diabetes in WFS versus pediatric patients with autoimmune T1D.

## Materials and methods

The study protocol was approved by the University Bioethics Committee at the Medical University in Lodz, Poland (RNN/133/10/KE). Patients and/or their parents gave written informed consent for participation in the study.

CGM was performed in two groups of patients: patients with WFS (*n* = 7; all females) and a 3:1 matched group of patients with T1D. Diagnosis of WFS was confirmed by direct sequencing of the *WFS1* gene and/or multiplex ligation-dependent probe amplification (MLPA; SALSA MLPA P163 GJB-WFS1 probemix, MRC-Holland, The Netherlands), as described previously [16].

The T1D group was selected using propensity score matching from all available females who underwent CGM procedures in pediatric diabetology centers in Lodz or Gdansk (*n* = 75). Propensity score matching was performed for age, duration of diabetes, glycated hemoglobin level, and average daily insulin dose. The resulting group of 21 girls was used in the analysis. All patients had diabetes mellitus recognized according to WHO criteria. Autoantibodies specific for T1D were detected only in patients with T1D. All seven patients with WFS and 19 of 21 patients with T1D were treated with continuous subcutaneous intensive insulin therapy. HbA1c was determined by high-performance liquid chromatography (HPLC) using the *Bio*-*Rad VARIANT*™* Hemoglobin A1c Program* (*Bio*-*Rad Laboratories, Inc*. Hercules, CA, USA) with its values represented as percentages and mmol/mol.

In all studied patients, a 72-h record of CGM using an iPro2 device (Medtronic MiniMed, Inc., Northridge, CA, USA) was generated. CGM sensor insertion was performed in the diabetes clinic by trained study personnel.

For analysis of the results obtained within at least one complete day, the application “GlyCulator” was used [17], which is available online (www.pediatria.umed.pl/team/glyculator). The following glycemic variability indices were evaluated in all patients: mean, standard deviation (SD), coefficient of variation (CV), M100 index, and J index as the measures of stability and quality of glycemic control; percentages of results above values of 126 and 180 mg/dL and below 70 and 54 mg/dL; and continuous overall net glycemic action (CONGA) [17]. CONGA values represent the SD of differences between the current observation and the observation n hours prior (i.e., CONGA1h = 1 h prior, CONGA2h = 2 h prior, CONGA4h = 4 h prior, CONGA6h = 6 h prior) [5].

In addition, C-peptide serum levels were estimated by applying the commercially available electrochemiluminescence method (ECLIA) (Roche Diagnostics GmbH, Germany). Fasting C-peptide levels from healthy individuals ranged from 1.1 to 4.4 ng/mL, and the detection limit has been assessed as 0.01 ng/mL for the assay.

### Statistical analysis

Normally distributed data were represented using means with standard deviations and were compared using Welch’s *t* test. Non-normally distributed variables were presented as medians with lower and upper quartile boundaries and compared between the groups using the Mann–Whitney *U* test. Results with *p* levels <0.05 were considered as statistically significant. Analyses were performed using Statistica 10.0 PL software (Statsoft, Tulsa, OK, USA).

## Results

The patients from the two study groups were matched based on sex and age (~13.5 years) and had similar HbA1c levels (~7.8 %), insulin requirements (~0.7 U/kg), insulin bolus/basal ratio, and long-term duration of diabetes (~8 years). However, WFS patients showed a significantly higher C-peptide level than T1D patients (*p* = 0.006). Characteristics of the study groups are shown in Table 1.

**Table 1:** Clinical characteristics of matched WFS and T1D patients

	WFS		T1D		*p* level
	*N*	Mean ± SD	*N*	Mean ± SD	
Age (years)	7	13.58 ± 4.12	21	13.85 ± 2.92	0.849
Diabetes duration (years)	7	8.08 ± 3.80	21	8.20 ± 3.10	0.933
HbA1c (%NGSP/mmol/mol IFCC)	7	7.80 ± 1.05/58.70 ± 11.90	21	7.76 ± 1.07/61.30 ± 12.00	0.943
Insulin requirement (U/kg of body weight)	7	0.68 ± 0.19	21	0.74 ± 0.31	0.611
Insulin bolus/basal ratio	7	0.70 ± 0.38	21	0.66 ± 0.12	0.774
C-peptide (ng/mL)	7	0.31 ± 0.20	21	0.04 ± 0.04	**0.006**

*p* value below 0.05 is marked in bold

*T1D* type 1 diabetes, *WFS* Wolfram syndrome, *SD* standard deviation

**Fig. 1:**
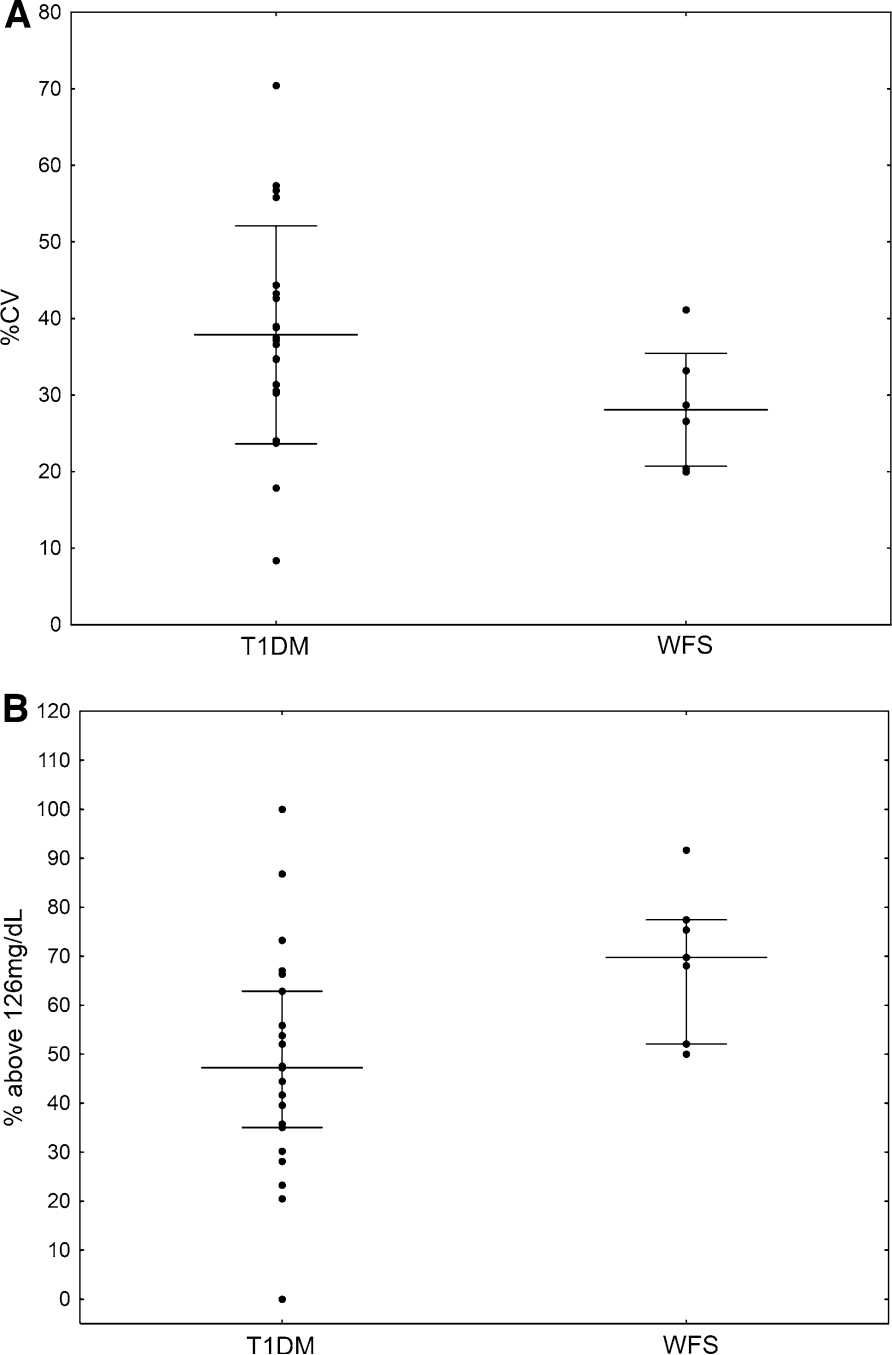
Comparison of percentage coefficient of variation (%CV) (**a**) and percentage of values above 126 mg/dL (**b**) in CGM between patients with type 1 diabetes (T1D) and Wolfram syndrome (WFS)

**Fig. 2:**
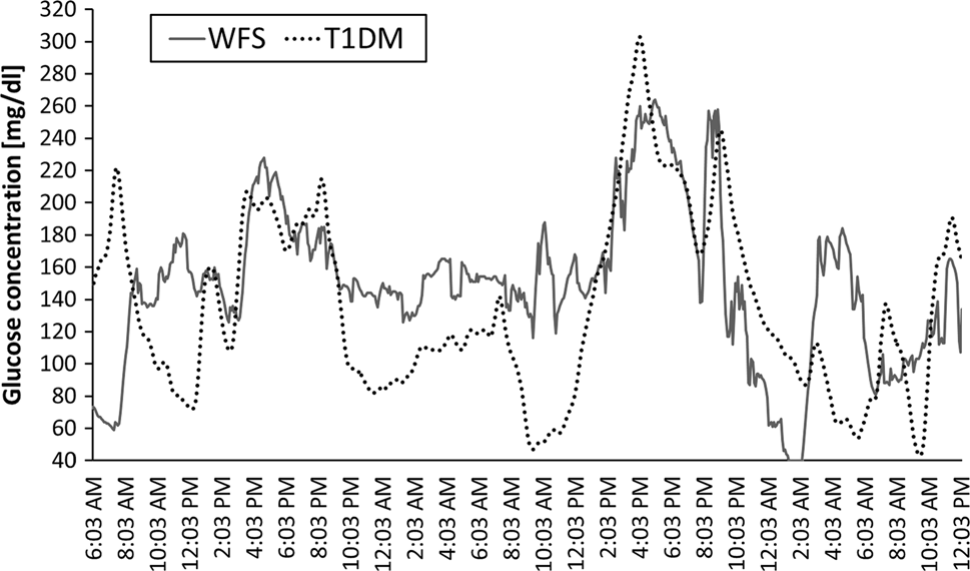
Differences in glycemic variability in CGM records from a patient with type 1 diabetes (*T1D* *dashed line*, average glycemia = 134.03 ± 51.26 mg/dL) and a patient with Wolfram syndrome (*WFS* *solid line*, average glycemia = 148.38 ± 49.78 mg/dL)

Comparisons of the CGM parameters showed that the %CV of glycemia (used to allow standardized comparisons between patients with different levels of mean glycemia) was significantly lower in WFS patients (28.08 ± 7.37) compared to T1D patients (37.87 ± 14.24, *p* = 0.029) (Fig. 1a). A tendency toward a lower SD, as the simplest tool for the assessment of glycemic variability, was found in patients with WFS (41.89 ± 10.34) as compared to T1D (52.40 ± 21.54, *p* = 0.099). All analyzed CGM parameters are summarized in Table 2. Figure 2 shows the differences in glycemic variability between a WFS patient and a T1D patient based on two CGM records which are representative for all studied patients.

**Table 2:** Comparison of CGM glycemic variability parameters between the study groups

Parameter	WFS group (Mean ± SD)	T1D group (Mean ± SD)	*p* level
Mean	150.28 ± 14.41	138.21 ± 29.38	0.166
Standard deviation	41.89 ± 10.34	52.40 ± 21.54	0.099
%CV	28.08 ± 7.37	37.87 ± 14.24	**0.029**
M100 index	12.18 ± 4.88	15.58 ± 12.22	0.305
J index	37.20 ± 6.81	38.16 ± 17.34	0.837
CONGA1h	34.29 ± 7.72	39.10 ± 14.26	0.273
CONGA2h	47.04 ± 9.52	58.06 ± 22.03	0.079
CONGA4h	54.96 ± 11.92	74.12 ± 28.74	**0.020**
CONGA6h	55.99 ± 10.58	80.26 ± 35.05	**0.009**

*p* values below 0.05 are marked in bold

*%CV* percentage coefficient of variation, *CONGA* continuous net glycemic action, *T1D* type 1 diabetes, *WFS* Wolfram syndrome

Following the above comparisons, we assessed CONGA parameters. CONGA4h and GONGA6h were significantly lower in patients with WFS (54.96 ± 11.92 and 55.99 ± 10.58, respectively) than in patients with T1D (74.12 ± 28.74, *p* = 0.020 and 80.26 ± 35.05, *p* = 0.009, respectively). CONGA2h showed a similar pattern but did not reach statistical significance (*p* = 0.079) (Table 2).

In WFS patients, the percentage of values above 126 mg/dL was 69.79 (52.08–77.43), whereas in T1D patients, it was significantly lower—47.22 (35.07–62.85, *p* = 0.018) (Fig. 1b). Curiously, there was a tendency toward a smaller percentage of measurements below 70 mg/dL in the WFS group [0 (0–7.29) %] compared to the T1D group [6.25 (0–18.06) %, *p* = 0.122]. Similar differences were noted for the percentage of nighttime hypoglycemic events, but the difference was not statistically significant (*p* = 0.148). All analyzed percentages are shown in Table 3.

**Table 3:** Comparison of percentages of CGM measurements between the study groups

	WFS group [median (25–75 %)]	T1D group [median (25–75 %)]	*p* level
% Above 126 mg/dL (7 mmol/l)	69.79 (52.08–77.43)	47.22 (35.07–62.85)	**0.018**
% Above 180 mg/dL (10 mmol/l)	19.10 (18.40–35.07)	22.92 (12.50–30.9)	0.614
% Below 70 mg/dL (3.89 mmol/l)	0 (0–7.29)	6.25 (0–18.06)	0.122
% Below 54 mg/dL (3 mmol/l)	0 (0–1.74)	0 (0–2.08)	0.816
% Of nighttime hypoglycemia [<70 md/dl (<3.89 mmol/l)]	0 (0–3.52)	3.04 (0.82–4.63)	0.148

*p* value below 0.05 is marked in bold

*T1D* type 1 diabetes, *WFS* Wolfram syndrome

## Discussion

In this study, glucose variability among diabetic patients with WFS was evaluated for the first time. This condition is rare among patients with diabetes [18]. In the Polish National Registry, we found 15 patients with diabetes and WFS. For this study, we were able to recruit seven patients from the entirety of Poland, and we compared these with a larger number of patients with T1D (1:3) that we matched using an objective statistical tool. Based on this approach, and using the CGM system, we found lower glycemic variability in pediatric patients with WFS in comparison with T1D patients. Furthermore, an increased percentage of hyperglycemia above 126 mg/dL in WFS patients was also noted. To date, efforts to evaluate the degree of metabolic control of diabetes in WFS relied on the measurement of HbA1c level, which is strongly linked to a risk of chronic complications, frequency of ketoacidosis and partial remission, and episodes of hypoglycemia. However, the literature has reported conflicting results. Some studies demonstrated lower daily insulin requirements and better glycemic control in patients with WFS as compared to T1D [14]. Rohayem et al. [15] in a large cohort of patients noted only a higher frequency of ketoacidosis at diabetes onset for T1D than for WFS patients, with a no significant differences in daily insulin dose and clinical remission.

Despite longer periods of hyperglycemia in our study, it should be noted that the glycemic variability of the WFS group was in fact lower than that of the T1D group. Although data on glycemic variability are far from conclusive, there is growing evidence that glycemic variability plays a role in the formation of reactive oxygen species and may increase the rates of diabetic complications [19, 20]. The reasons behind the lower glycemic variability in WFS patients may be manifold—from more conservative treatment regimens ordered by their doctors to more efficient counter-regulatory mechanisms. We did our best to mitigate the impact of patient selection through the matching procedure and picked groups with similar HbA1c levels and insulin requirements to assure that our measurements of variability would be free from these confounders. It would thus seem more likely that the lower glycemic variability we observed in patients with WFS may be an effect of an intrinsic biological factor rather than of diabetes self-management.

Rohayem et al. [15] reported a lower prevalence of microvascular complications and a higher frequency of severe hypoglycemia in WFS patients in comparison with T1D patients. In our study, a tendency toward a reduced frequency of hypoglycemia episodes below 70 mg/dL in WFS patients in comparison with T1D patients was observed, whereas the incidence of severe hypoglycemia (below 54 mg/dL) was similar in both study groups.

The observed differences may be related to more efficient secretion of endogenous insulin in WFS than in T1D patients. A persistent insulin secretory reserve could also explain the protection of our WFS patients from significant changes in glycemia levels recorded during CGM. Differences in insulin secretion measured by C-peptide level between these types of diabetes were already reported by some researchers [21]. Other studies reported different results concerning severe insulin deficiency in both diseases [22, 23]. However, we confirmed there is preserved residual insulin secretion (based on the concentrations of C-peptide) in WFS patients, but not in T1D individuals.

On the other hand, we would like to emphasize that the reduced incidence of hypoglycemia with an increased percentage of hyperglycemia could be also related to the preserved permanent glucagon response in WFS patients. This hypothesis is consistent with the results obtained in WFS animal models by Ishihara et al. [12] who demonstrated progressive loss of β-cells in mutant mice while the α-cells were completely preserved. In contrast, other studies have reported that α-cell function and the glucagon response are impaired in patients with T1D [24, 25].

Unfortunately, we were not able to measure plasma glucagon levels of the patients at the time of the CGM study, which would be a valuable complement to the presented results. Because of this limitation of the study, our observations on the importance of glucagon can be only speculative.

The high frequency of hyperglycemia observed in our study in WFS patients is even more concerning in light of reports of the long-term adverse impact of hyperglycemia on the brain of diabetic patients. Results obtained by Mauras et al. [26] using high-resolution structural magnetic resonance imaging and the CGM system suggest the presence of disturbances in the structure of gray and white matter of the brain in children with T1D associated with chronic hyperglycemia. Prolonged hyperglycemia can be especially damaging for WFS patients who have many neurodegenerative features detected by neuroimaging studies [27, 28]. Therefore, every extensive effort to limit the episodes of hyperglycemia among subjects with WFS should be recommended and this information is needed for clinicians who deal with these patients.

In conclusion, our study showed for the first time that patients with WFS have smaller glycemic variability than individuals with T1D, and this is probably associated with persistent residual insulin secretion in WFS.

## References

[1] Langendam M, Luijf YM, Hooft L, Devries JH, Mudde AH, Scholten RJ (2012). Continuous glucose monitoring systems for type 1 diabetes mellitus. Cochrane Database Syst Rev.

[2] Chase HP, Beck RW, Xing D, Tamborlane WV, Coffey J, Fox LA, Ives B, Keady J, Kollman C, Laffel L, Ruedy KJ (2010). Continuous glucose monitoring in youth with type 1 diabetes: 12-month follow-up of the Juvenile Diabetes Research Foundation continuous glucose monitoring randomized trial. Diabetes Technol Ther.

[3] Garg S, Jovanovic L (2006). Relationship of fasting and hourly blood glucose levels to HbA1c values: safety, accuracy, and improvements in glucose profiles obtained using a 7-day continuous glucose sensor. Diabetes Care.

[4] Hommel E, Olsen B, Battelino T, Conget I, Schütz-Fuhrmann I, Hoogma R, Schierloh U, Sulli N, Gough H, Castañeda J, de Portu S, Bolinder J, SWITCH Study Group (2014). Impact of continuous glucose monitoring on quality of life, treatment satisfaction, and use of medical care resources: analyses from the SWITCH study. Acta Diabetol.

[5] Patton SR, Williams LB, Eder SJ, Crawford MJ, Dolan L, Powers SW (2011). Use of continuous glucose monitoring in young children with type 1 diabetes: implications for behavioral research. Pediatr Diabetes.

[6] Brancato D, Saura G, Fleres M, Ferrara L, Scorsone A, Aiello V, Di Noto A, Spano L, Provenzano V (2013). Prognostic accuracy of continuous glucose monitoring in the prediction of diabetes mellitus in children with incidental hyperglycemia: receiver operating characteristic analysis. Diabetes Technol Ther.

[7] Steck AK, Dong F, Taki I, Hoffman M, Klingensmith GJ, Rewers MJ (2014). Early hyperglycemia detected by continuous glucose monitoring in children at risk for type 1 diabetes. Diabetes Care.

[8] Borowiec M, Mysliwiec M, Fendler W, Antosik K, Brandt A, Malecki M, Mlynarski W (2011). Phenotype variability and neonatal diabetes in a large family with heterozygous mutation of the glucokinase gene. Acta Diabetol.

[9] Barrett TG, Bundey SE, Macleod AF (1995). Neurodegeneration and diabetes: UK nationwide study of Wolfram (DIDMOAD) syndrome. Lancet.

[10] Minton JAL, Rainbow LA, Ricketts Ch, Barrett TG (2003). Wolfram syndrome. Rev End Metabol Dis.

[11] Karasik A, O’Hara C, Srikanta S, Swift M, Soeldner JS, Kahn CR, Herskowitz RD (1989). Genetically programmed selective islet β-cell loss in diabetic subjects with Wolfram’s syndrome. Diabetes Care.

[12] Ishihara H, Takeda S, Tamura A, Takahashi R, Yamaguchi S, Takei D, Yamada T, Inoue H, Soga H, Katagiri H, Tanizawa Y, Oka Y (2004). Disruption of the WFS1 gene in mice causes progressive beta-cell loss and impaired stimulus-secretion coupling in insulin secretion. Hum Mol Genet.

[13] Yamada T, Ishihara H, Tamura A, Takahashi R, Yamaguchi S, Takei D, Tokita A, Satake C, Tashiro F, Katagiri H, Aburatani H, Miyazaki J, Oka Y (2006). WFS1-deficiency increases endoplasmic reticulum stress, impairs cell cycle progression and triggers the apoptotic pathway specifically in pancreatic beta-cells. Hum Mol Genet.

[14] Cano A, Molines L, Valéro R, Simonin G, Paquis-Flucklinger V, Vialettes B, French Group of Wolfram Syndrome (2007). Microvascular diabetes complications in Wolfram syndrome [diabetes insipidus, diabetes mellitus, optic atrophy, and deafness (DIDMOAD)]. Diabetes Care.

[15] Rohayem J, Ehlers C, Wiedemann B, Holl R, Oexle K, Kordonouri O, Salzano G, Meissner T, Burger W, Schober E, Huebner A, Lee-Kirsch MA, Wolfram Syndrome Diabetes Writing Group (2011). Diabetes and neurodegeneration in Wolfram syndrome: a multicenter study of phenotype and genotype. Diabetes Care.

[16] Zmyslowska A, Borowiec M, Antosik K, Szalecki M, Stefanski A, Iwaniszewska B, Jedrzejczyk M, Pietrzak I, Mlynarski W (2011). Wolfram syndrome in the polish population: novel mutations and genotype-phenotype correlation. Clin Endocrinol (Oxf).

[17] Czerwoniuk D, Fendler W, Walenciak L, Mlynarski W (2011). GlyCulator: a glycemic variability calculation tool for continuous glucose monitoring data. J Diabetes Sci Technol.

[18] Zmysłowska A, Borowiec M, Fendler W, Jarosz-Chobot P, Myśliwiec M, Szadkowska A, Młynarski W (2014). The prevalence of Wolfram syndrome in a paediatric population with diabetes. Endokrynol Pol.

[19] Monnier L, Mas E, Ginet C, Michel F, Villon L, Cristol JP, Colette C (2006). Activation of oxidative stress by acute glucose fluctuations compared with sustained chronic hyperglycemia in patients with type 2 diabetes. JAMA.

[20] Kilpatrick ES, Rigby AS, Atkin SL (2008). A1C variability and the risk of microvascular complications in type 1 diabetes: data from the diabetes control and complications trial. Diabetes Care.

[21] Fishman L, Ehrlich RM (1986). Wolfram syndrome: report of four new cases and a review of literature. Diabetes Care.

[22] Medlej R, Wasson J, Baz P, Azar S, Salti I, Loiselet J, Permutt A, Halaby G (2004). Diabetes mellitus and optic atrophy: a study of Wolfram syndrome in the Lebanese population. J Clin Endocrinol Metab.

[23] Pecheur A, Barrea T, Vandooren V, Beauloye V, Robert A, Lysy PA (2014). Characteristics and determinants of partial remission in children with type 1 diabetes using the insulin-dose-adjusted A1C definition. J Diabetes Res.

[24] Sherr J, Xing D, Ruedy KJ, Beck RW, Kollman C, Buckingham B, White NH, Fox L, Tsalikian E, Weinzimer S, Arbelaez AM, Tamborlane WV, Diabetes in Children Network (2013). Lack of association between residual insulin production and glucagon response to hypoglycemia in youth with short duration of type 1 diabetes. Diabetes Care.

[25] Greenbaum CJ, Prigeon RL, D’Alessio DA (2002). Impaired beta-cell function, incretin effect, and glucagon suppression in patients with type 1 diabetes who have normal fasting glucose. Diabetes.

[26] Mauras N, Mazaika P, Buckingham B, Weinzimer S, White NH, Tsalikian E, Hershey T, Cato A, Cheng P, Kollman C, Beck RW, Ruedy K, Aye T, Fox L, Arbelaez AM, Wilson D, Tansey M, Tamborlane W, Peng D, Marzelli M, Winer KK, Reiss AL; for the Diabetes Research in Children Network (DirecNet) (2014) Longitudinal assessment of neuroanatomical and cognitive differences in young children with type 1 diabetes: association with hyperglycemia. Diabetes. pii: DB_141445

[27] Hershey T, Lugar HM, Shimony JS, Rutlin J, Koller JM (2012). Early brain vulnerability in Wolfram syndrome. PLoS One.

[28] Zmyslowska A, Malkowski B, Fendler W, Borowiec M, Antosik K, Gnys P, Baranska D, Mlynarski W (2014). Central nervous system PET-CT imaging reveals regional impairments in pediatric patients with Wolfram syndrome. PLoS One.

